# POLICY SERIES: NEW FAMILY CAREGIVING RESEARCH TO SUPPORT POLICY AND PRACTICE CHANGE

**DOI:** 10.1093/geroni/igz038.2739

**Published:** 2019-11-08

**Authors:** Jean C Accius, Heather Young

**Affiliations:** 1 AARP, Washington, District of Columbia, United States; 2 Betty Irene Moore School of Nursing - UC Davis, Sacramento, California, United States

## Abstract

Families are the most important source of support to older adults. Today, there is growing recognition of the escalating complexity of family caregiving. Family caregivers are increasingly carrying out health-related tasks with little training or preparation, as well as continuing to provide the majority of long-term services and supports (LTSS) at home. Providing care in the context of rapidly changing health care and LTSS systems can have a significant impact on the family members who provide care and take a significant toll, emotionally, physically, and financially. Studies commonly show that family caregivers report learning complex tasks by trial and error and worry about making a mistake. This symposium highlights new caregiving research from the AARP Public Policy Institute. The first paper will present new research on the increasing complexity of the challenges facing family caregivers, such as managing multiple medications, wound care, and interaction with the health care system. The second paper will present new data on the economic value of the unpaid contributions of family caregivers in the United States. The third paper will examine the growing importance of family caregiving on the public policy agenda, and describe recent policy developments that recognize and explicitly support caregiving families. Recommendations for policy and practice change to address caregiving needs will be examined in all papers. Our discussant will identify key implications from this research for policymakers and practitioners, and potential drivers for developing a better system of family support at the federal and state levels.

